# Cumulative live birth rates of patients in the Peruvian Andes according to the POSEIDON criteria: a single-center retrospective analysis

**DOI:** 10.5935/1518-0557.20200107

**Published:** 2021

**Authors:** Luis Vargas-Tominaga, Andrea Vargas, Fiorella Alarcón, Andrea Medina, Maritza Gómez, Katherine Bejar

**Affiliations:** 1 Centro de Fertilidad y Ginecología del Sur, Cusco, Peru

**Keywords:** POSEIDON criteria, low ovarian response, assisted reproductive technology, cumulative live birth rate

## Abstract

**Objective:**

To identify low prognosis *in-vitro* fertilization (IVF) patients treated at Centro de Fertilidad y Ginecología del Sur (CFGS) based on the POSEIDON criteria.

**Methods:**

This retrospective study included 412 IVF patients and assessed them based on the POSEIDON criteria to describe the cumulative live birth rates seen in each group.

**Results:**

13.1% of patients met the POSEIDON criteria, and the proportion of cases in POSEIDON groups 1, 2, 3 and 4 were 20.4%, 31.5%, 14.8%, and 33.3%, respectively. The cumulative live birth rate for the individuals meeting the POSEIDON criteria was 25.9%, while for patients in POSEIDON groups 1, 2, 3, and 4 the rates were 45.5%, 29.4%, 25.0%, and 11%, respectively. The differences were not statistically significant due to the small number of cases.

**Conclusions:**

Patients belonging to the four POSEIDON groups were described at CFGS. Age and number of retrieved mature oocytes were determining parameters in the prognosis of pregnancy in IVF/ICSI patients.

## INTRODUCTION

Among infertile patients, women with low ovarian response (LOR) to exogenous gonadotropins are a constant challenge in clinical management. As age increases, the number of follicles decreases, chromosomal abnormalities and dysfunction of the oocyte cytoplasm increase, and the possibility of achieving pregnancy through natural means or assisted reproduction decreases. Younger women reportedly have greater euploid embryo rates and better cumulative pregnancy rates (CPR) (Esteves et al., 2019a; Franasiak *et al*., 2014; Ata *et al*., 2012).

CPR in LOR patients varies in the literature, and one of the reasons is the heterogeneity in the definition of this group of patients. Most reports on LOR consider the number of oocytes obtained in aspiration as the most important parameter, while others see age as the most relevant criterion (Sunkara *et al*., 2011; De Geyter *et al.*, 2015). Nonetheless, more than forty criteria have been used in the definition of LOR (Esteves *et al*., 2019b).

The European Society for Human Reproduction and Embryology (ESHRE), in an attempt to unify and outline standards for LOR, held a consensus meeting in the city of Bologna, Italy, in 2010. The consensus produced the first opportunity to order this group of patients, although a number of researchers indicated that it failed to eliminate the variability in the diagnosis of LOR stemmed from different studies. Additionally, the consensus did not consider female patient age or oocyte competence in terms of embryo aneuploidy rate (POSEIDON Group, 2016).

The POSEIDON (Patient-Oriented Strategies Encompassing Individualize D Oocyte Number) criteria intends to group patients based on oocyte quality (age) and number of oocytes (ovarian reserve) (Humaidan *et al*., 2016; Bühler *et al*., 2020). Euploidy rate is based on the age of the female patient, not from the number of obtained blastocysts (Ata *et al*., 2012). More metaphase II (MII) oocytes retrieved means more euploid blastocysts. However, the euploidy rate is consistent across the number of MII oocytes retrieved (Colamaria et al., 2015).

POSEIDON stratification considers age and ovarian reserve as determining factors and introduces the concept of “low prognosis” patients. The POSEIDON criteria serve as a guide to set up strategies in ovarian stimulation cycles and patient management, with the aim of obtaining at least one euploid embryo for transfer.

The present study used the POSEIDON stratification criteria to find low prognosis patients at Centro de Fertilidad y Ginecología del Sur (CFGS), a center in the Peruvian Andes at an altitude of more than 3,300 meters above mean sea level (AMSL).

## MATERIALS AND METHODS

In this retrospective study, we reviewed charts and records of *in-vitro* fertilization (IVF) and intracytoplasmic sperm injection (ICSI) procedures performed in patients with autologous oocytes conducted at CFGS from June 2009 to March 2020. We included consecutively all IVF/ICSI cycles and excluded only patients whose follow-up information could not be found. CFGS is a fertility center located in the city of Cusco, in the Peruvian Andes, at an altitude of 3,330 m AMSL. The institutional review board approved the study and all patients consented to having their data used in the study.

**Figure 1:**
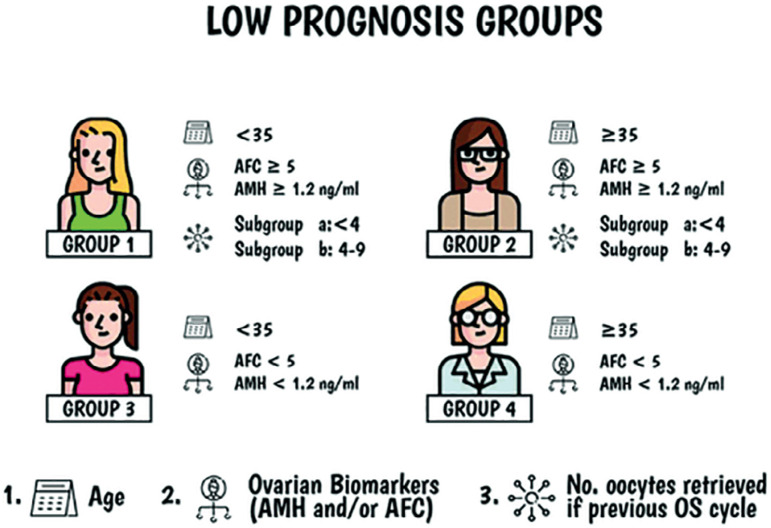
Low Prognosis Group. Modified from: POSEIDON group. Fertil Steril. 2016;105:1452–3; Humaidan et al. F1000Res. 2016; 5: 2911; Esteves et al. Front Endocrinol. 2019b; 10: 814.

Antral follicular count (AFC) was defined as the number of follicles of 2-9 mm in diameter. AFC is performed 2-5 days after the start of the menstrual cycle via vaginal ultrasound, in the three months previous to the IVF/ICSI cycle. The POSEIDON stratification criteria defines “low prognosis” patients in the following four groups (Figure 1): Group 1: age < 35, AFC ≥ 5 or anti-Müllerian hormone (AMH) ≥ 1.2 ng/ml and the number of oocytes retrieved ≤ 9 in the previous cycle. Group 2: age ≥ 35, AFC ≥ 5 or AMH ≥ 1.2 ng/ml and the number of oocytes retrieved ≤ 9 in the previous cycle. Group 3: age < 35, AFC < 5 or AMH ˂ 1.2 ng/ml. Group 4: age ≥ 35, AFC < 5 or AMH ˂ 1.2 ng/ml. In groups 1 and 2, it establishes a subgroup “a” when ˂ 4 oocytes were retrieved in the previous cycle, and a group “b”, when 4 to 9 oocytes were retrieved in the previous cycle (POSEIDON group, 2016). We decided to use the AFC instead of AMH levels, since this is a parameter that we have recorded for all of our patients.

Ovarian stimulation was performed with human menopausal gonadotropin (HMG) or recombinant FSH (rFSH), in association with GnRH analogues, using agonists (a-GnRH) in long or short protocols, or antagonists (ant-GnRH) in flexible protocols. We followed follicular development through vaginal ultrasound, and aspiration was scheduled 36 hours after triggering with urinary or recombinant human chorionic gonadotropin (hCG). Follicular aspiration was performed under sedation with a single lumen needle and vaginal ultrasound guidance. Luteal support was simultaneously started with 600 to 800 mg of micronized progesterone daily (Utrogestan, Ferring^TM^ or Geslutin PNM, Tecnofarma^TM^) or progesterone ring (Fertiring, ABL Pharma^TM^), vaginally, or intramuscular progesterone, 50 mg daily.

Life-Global™ culture medium was used for gamete manipulation, and continuous embryo culture was carried out in incubators (Thermo Scientific^TM^3111 Water-Jacketed CO_2_ incubator, Astec^TM^ EC-6S or K-System^TM^ G210 InviCell) at 9.0% of CO_2_ concentration and 37ºC of temperature. In IVF patients, the retrieved oocytes were placed in petri dishes with culture medium, containing 100,000 to 200,000 capacitated sperms. In ICSI cases, sperm was prepared using density gradients and selected per swim-out in 5µl drops on the ICSI plate. At 16h, fertilization was verified and the embryos were placed in culture medium to the cleavage or blastocyst stage. When the number of fertilized oocytes was >5, we extended culture to the blastocyst stage without changing the culture medium. Embryo transfer was performed under abdominal ultrasound guidance, with a full bladder, using a flexible catheter; the patients would then rest for 45 minutes.

Surplus embryos were vitrified (Kuwayama *et al*., 2005) and stored in liquid nitrogen. In subsequent frozen embryo transfers (FET), patients took oral estradiol valerate (Progynova, Bayer^TM^) in doses increased gradually from 2 to 12 mg daily until they achieved endometrial thickness ≥ 6 mm. Luteal support was initiated 3 or 5 days before embryo transfer (ET), depending of the embryo development stage (EDS).

After 13 to 15 days of ET, the patients were tested for β-hCG levels. If the result was positive, a vaginal ultrasound was performed 1 or 2 weeks after the test. Clinical pregnancy was diagnosed for patient with a gestational sac with an active embryo. Some patients remained at our clinic for pre-natal care and delivery, while others were contacted by phone to monitor pregnancy progress.

Live birth (LB) was defined as a neonate showing signs of life, irrespective of gestational age, as defined by the World Health Organization (2010). The cumulative live birth rate (CLBR) within one complete IVF/ICSI treatment cycle was defined as the probability of a LB from ovarian stimulation, including all embryo transfers (fresh and frozen) from one stimulation cycle. Live birth rate (LBR) only considers the result from the stimulation cycle.

Statistical significance of the found differences was analyzed with the Chi-square test or Fisher’s exact test.

## RESULTS

We performed complete IVF/ICSI cycles in 421 patients, including all embryo transfers (fresh and frozen), and excluded nine patients lost during the follow-up. Table 1 shows baseline and treatment characteristics in POSEIDON and NON-POSEIDON groups.

**Table 1:** Baseline and treatment characteristic in POSEIDON and NON-POSEIDON groups

	POSEIDON	1a	1b	2a	2b	3	4	NON-POSEIDON
Cases	54	1	10	7	10	8	18	358
Age of female patient (*)	36.69 (35.64-37.73)	33.00	33.00 (32.33-33.67)	39.00 (35.80-42.20)	36.70 (35.63-37.77)	32.13 (30.91-33.34)	40.06 (38.81-41.30)	34.44 (33.99-34.90)
Infertility duration (years) (*)	5.14 (4.06-6.21)	3.00	6.20 (2.93-9.47)	6.71 (3.35-10.08)	6.60 (3.54-9.66)	3.88 (2.50-5.25)	3.53 (1.57-5.50)	5.13 (4.74-5.52)
AFC (*)	7.75 (5.71-9.78)	20	14.50 (7.37-21.63)	5.86 (4.87-6.85)	11.80 (7.02-16.58)	3.50 (2.61-4.39)	2.94 (2.48-3.41)	13.53 (12.84-14.22)
GnRH analogues (a-GnRH/ant-GnRH/without)	14/34/6	0/1/0	4/6/0	4/3/0	1/8/1	2/4/2	3/12/3	77/278/3
Gonadotropin dose (IU) (*)	1883.33 1647.81-2118.86)	2400.00	1967.50 (1646.80-2288.20)	2403.57 (1739.81-3067.33)	2242.50 (1850.32-2634.68)	2164.29 (1099.89-3228.68)	1345.59 (890.66-1800.52)	2121.34 (2062.49-2180.18)
MII oocytes (*)	5.08 (3.86-6.30)	14	9.30 (5.84-12.76)	3.14 (0.61-5.67)	7.10 (4.11-10..09)	2.57 (1.39-3.75)	2.56 (1.70-3.41)	8.45 (7.93-8.97)
Day of transfer (cleavage/blastocyst)	40/14	0/1	3/7	6/1	8/2	8/0	15/3	231/127
Embryos transferred (*)	2.35 (2.04-2.67)	2.00	2.80 (2.24-3.36)	2.00 (1.08-2.92)	3.20 (2.32-4.08)	2.25 (1.38-3.12)	1.89 (1.38-2.40)	2.68 (2.60-2.77)

* 95% confidence interval

Fifty-four (13.1%) of 412 patients met the POSEIDON criteria, and 358 (86.9%) were included in the NON-POSEIDON group. Patients meeting the POSEIDON criteria were further stratified into groups 1, 2, 3 and 4, in the following respective proportions: 20.4%, 31.5%, 14.8%, and 33.3% (Table 2). Individuals in subgroups 1a and 1b amounted to 1.9% and 18.5% of the subjects in the POSEIDON group, while subjects in subgroup 2a and 2b accounted for 13.0% and 18.5% of the patients in the POSEIDON group, respectively.

**Table 2:** Distribution and CLBR in POSEIDON groups

Group	n	% a,b	% groups	MC	LB	FET	MC-FET	LB-FET	CLBR a,b	CLBR groups	*p* value
1a	1	1.9%	20.4%	0	0	0	0	0	0.0%	45.5% (5/11)	0.337
1b	10	18.5%		0	5	1	0	0	50.0%		
2a	7	13.0%	31.5%	0	1	1	0	0	14.3%	29.4% (5/17)	1.000
2b	10	18.5%		0	4	1	0	0	40%		
3	8		14.8%	0	2	0	0	0		25.0% (2/8)	1.000
4	18		33.3%	0	2	1	0	0		11.1% (2/18)	0.069
Not Poseidon	358			16	102	55	2	15		32.7% (117/358)	0.351

n, number of cases; % a,b, percentage of cases in groups a and b; % groups, percentage of cases in groups 1-4; MC, miscarriage; LB, live birth; FET, frozen embryo transfer; MC-FET, miscarriage after FET; LB-FET, LB from FET; CLBR a,b, cumulative live birth rate in groups a and b; CLBR groups, CLBR in POSEIDON groups.

In fresh transfers, the POSEIDON group had 54 ET, 14 live births and no miscarriages, while in the NON-POSEIDON group, 358 patients had ET leading to 102 live births and 16 miscarriages. FET was performed in four patients in the POSEIDON group, with negative results in all transfers. In the NON-POSEIDON group, we performed FET in one attempt in 53 patients, in two attempts in 10, in three attempts in 3, and in four attempts in 1. We achieved 15 live births and 2 patients had miscarriages (Table 2).

CLBR in the entire POSEIDON group was 25.9%. CLBR by groups was 45.5% in group 1, while lower CLBR was seen in group 2 and 3, with 29.4% and 25.0%, respectively; group 4 had the lowest CLBR, at 11.1%. In the NON-POSEIDON group, CLBR was 32.7% (Table 2).

## DISCUSSION

The Bologna consensus defined LOR when two of the following three criteria are met: advanced age (≥40 years), previous poor response cycle (≤3 oocytes after conventional stimulation protocol), or an abnormal ovarian reserve test (AFC less than 5-7 or AMH less than 0.5-1.1 ng/ml) (Ferraretti et al., 2011). The POSEIDON criteria introduced the concept of “low prognosis” according to age and ovarian reserve, considering 35 years of age and an AFC of 5 or an AMH level of 1.2 ng/ml as edge-points (POSEIDON group, 2016).

At CFGS, only 13.1% of IVF/ICSI patients met the POSEIDON criteria, unlike the 24.5% reported by Shi *et al*. (2019), the 31.5% by Li *et al*. (2019), and the 52.6% found by Seven *et al*. (2020). It is possible that our proportion of low prognosis patients is due to the fact that a large part of our IVF/ICSI patients are young (45.8% of IVF/ICSI patients at CFGS are under 35 years of age). In addition, many of our low prognosis patients seek donor eggs, since they provide a greater chance of pregnancy (44.6% of cycles at CFGS are performed with donor eggs) (Vargas *et al*., 2016).

At CFGS, groups 1, 2, 3 and 4 accounted for 20.4%, 31.5%, 14.8% and 33.3% of the patients (Table 1), i.e., two-thirds were older women. This distribution is different than the numbers reported in other studies and depends on the characteristics of the population seen at each center (Table 3). According to Abu-Musa, group 4 tends to be the group with the highest number of patients, accounting for about 55% of the patients, while group 3 amounts to about 10% (Abu-Musa *et al*., 2020). The study by Levi-Setti *et al*. (2019) included IVF/ICSI patients in whom 1-9 oocytes had been retrieved, instead of selecting patients who had had ≤ 9 oocytes in a previous cycle of ovarian stimulation and aspiration, as described in the POSEIDON criteria. However, important information can be derived, since 61.5% of the patients were in group 4, and only 6.9% were in group 1 (Levi-Setti *et al*., 2019).

**Table 3:** Distribution in POSEIDON groups

Author	Group 1	Group 2	Group 3	Group 4
Present study	20.4%	31.5%	14.8%	33.3%
Levi-Setti *et al*. 2019	6.9%	19.8%	11.7%	61.5%
Shi *et al*., 2019	24.9%	13.7%	24.3%	37.1%
Li *et al*., 2019	60.9%	24.5%	3.4%	11.2%

Shi *et al*. (2019) analyzed 18,455 cases of fresh IVF/ICSI cycles with ET and subsequent FET cycles, and strictly followed the POSEIDON criteria. The authors observed that half of the patients distributed homogeneously between groups 1 and 3 (24.9% and 24.3%, respectively), 13.7% were in group 2, and 37.1% were in group 4, i.e., almost two-thirds of the women had low ovarian reserve (Shi et al., 2019). Shi et al. (2019) also strictly followed the POSEIDON criteria, and found that 60.9% of 19,781 cases were in group 1, 24.5% in group 2, only 3.4% in group 3, and 11.2% in group 4. Contrary to Shi *et al*. (2019), this study included mostly from patients with adequate ovarian reserve.

In our study, CLBR in the entire POSEIDON group was 25.9%, and was better in group 1 (45.5), intermediate in group 2 and 3 (29.4% and 25.0%, respectively), and lower in group 4 (11.1%) (Table 2). Shi *et al*. (2019) reported higher CLBR in groups 1 and 3, which correspond to young women. Shi *et al*. (2019) considered oocyte quality as a more important factor, which is dependent on age. For individuals in group 2 (older women with good ovarian reserve), the authors recommended strategies aimed at oocyte quality rather than quantity: bringing the culture to blastocyst stage, achieving embryos with lower chances of aneuploidy and better candidates for implantation. Preimplantation genetic testing of aneuploidies (PGT-A), the selection of stimulation protocols aimed at improving oocyte quality, as well as the use of testosterone to improve follicular development, are alternatives to use in this group. For group 3, (young women with low ovarian reserve), recommendations include increasing the number of oocytes available and trying several cycles of ovarian stimulation instead of using high doses of gonadotropins (Shi *et al*., 2019). Li *et al*. (2019) found better results in group 1, intermediate outcomes in group 2, and poorer outcomes in groups 3 and 4. In contrast with Shi *et al*. (2019), the best results were dependent on the ovarian reserve rather than age. Li *et al*. (2019) did not find improvements in CLBR despite the change in ovarian stimulation protocol.

Leijdekkers *et al*. (2019) carried out a retrospective study with 551 patients using the POSEIDON criteria, considering an AMH level cut-off at 0.96 ng/ml, and calculated the CLBR in the four groups after several IVF/ICSI cycles during 18 months of observation. For purposes of comparison, we considered only the results obtained by Leijdekkers *et al*. (2019) in the first stimulation cycle, both fresh and FET. The authors found in the first cycle that younger individuals (groups 1 and 3) had better CLBR (Table 4). The authors looked into findings after 18 months of consecutive cycles and found that groups 1 and 3 had better CLBR (63% in group 1a, 67% in group 1b and 58% in group 3), with rates similar to patients with good prognoses, suggesting that poor response in young patients is possibly associated with decreased oocyte quality, with repeated cycles acting to overcome this condition (Leijdekkers *et al*., 2019).

**Table 4:** LBR and CLBR in POSEIDON groups

Author	Group 1	Group 2	Group 3	Group 4
Present study (CLBR)	45.5%	29.4%	25.0%	11.1%
Levi-Setti *et al*. (LBR)	27.1%	16.3%	24.0%	12.8%
Shi *et al*., 2019 (CLBR)	44.6%	24.5%	35.5%	12.7%
Li *et al*., 2019 (CLBR)	56.0%	30.9%	14.7%	6.6%
Leijdekkers *et al*., 2019 (CLBR)	39.2%	20.3%	29.2%	16.7%

Seven *et al*. (2020), in a retrospective study of 276 patients meeting the POSEIDON criteria undergoing fresh ET, found similar implantation and pregnancy rates across groups, with higher LBR seen groups 1 and 2. Among groups with lower ovarian reserve, patients in group 3 outperformed individuals in group 4 on LBR. POSEIDON stratification at CFGS showed age and ovarian reserve as critical factors, with similar effect on CLBR in groups 2 and 3 (29.4% and 25.0%, respectively). Group 1, with better age and ovarian reserve, CLBR was high (45.5%), and contrary to group 4, with both unfavorable parameters, CLBR was low (11.1%). The differences were not statistically significant on account of the small number of cases. However, trends were evident. Individuals meeting the POSEIDON criteria with better ovarian reserve had a greater number of mature oocytes and better CLBR, as observed in groups 1 and 2b, as well as in the NON-POSEIDON group (Table 1 and Table 2).

## CONCLUSION

The POSEIDON criteria allowed the identification of four groups of patients at CFGS. Age and number of retrieved mature oocytes were determining parameters in the prognosis of pregnancy in IVF/ICSI patients.
